# Monthly Prices of Substances Used in Pharmacy

**Published:** 1814-11

**Authors:** 


### *Monthly Prices of* SUBSTANCES *used in* PHARMACY,

**At Messrs. SELWAY and HENLEY's (Chemical and Pharmaceutical Laboratory,) 35, Upper Mary-le-bone Street, Portland Place.**

| | s. | d. |
|---|---|---|
| Acetum Scillæ .......... lb. | 2s. | 0d. |
| Abietis Resina .............. | 2 | 3 |
| Acaciæ Gummi, from 2s. 3d. to | 3 | 6 |

ACIDUM Aceticum, cong. 3s. 6d.—Benzoic. oz. 6s. 0d.—Oxalic. lb. 18s. 0d. —Nitric. 4s. 6d.—Nitros. 2s. 6d.—Muriatic. 0s. 9d.—Sulphuric. 0s. 6d. —Sulph. Arom. 5s 0d.

| | s. | d. |
|---|---|---|
| Alcohol sp. gr. 815 M. lb. | 4 | 6 |
| Æther sulphur. sp. gr. 789 | 9 | 0 |
| ——— rectificatus sp. gr. 744 | 11 | 0 |
| Ærugo ................ lb. | 6 | 6 |
| Aloes spicatæ extractum .... | 5 | 6 |
| —— vulgaris extractum .... | 4 | 6 |
| —— Barbadoes ............ | 5 | 0 |
| Alumen, 4d. exsiccatum .... | 0 | 8 |
| Ammoniæ Murias, 2s. 2d. to | 2 | 9 |
| ——— Carbonas ........ | 3 | 6 |
| Amygdalæ Amaræ, 2s. Dulc. | 5 | 8 |
| Ammoniacum, from 3s. 6d. to | 7 | 6 |
| Antimonii Sulphuretum .... | 1 | 0 |
| ——— Aurum. Sulph. .. | 6 | 0 |
| ——— Murias ........ | 3 | 0 |
| Antimonium Tartarizatum .. | 5 | 0 |
| Anthemidis Flores, 1s. 9d. to | 2 | 0 |
| Aqua Cinnamomi vera .... | 18 | 0 |
| —— Rosæ .............. | 5 | 6 |
| Aliæ Aquæ, from 2s. 6d. to | 3 | 6 |
| Arsenici Oxydum ........ lb. | 1 | 0 |
| Argenti Nitras ............ | 6 | 9 |
| Assafœtida Gummi-resina .. | 6 | 9 |
| Aurantii Cortex .......... | 2 | 6 |
| Balsamum Peruvianum ...... | 33 | 0 |
| ——— Tolutanum ...... | 15 | 0 |
| Benzoinum elect. .......... | 11 | 0 |
| Calamina præp. .......... | 0 | 3 |
| Calumbæ Radix .......... | 2 | 6 |
| Cambogia opt. ............ | 9 | 6 |
| Camphora ................ | 7 | 0 |
| Canellæ Cortex .......... | 3 | 4 |
| Cardamomi Semina opt..... | 12 | 0 |
| Cascarillæ Cortex .......... | 4 | 0 |
| Castoreum ............ unc. | 4 | 4 |
| Caryophilli .......... lb. | 11 | 0 |
| Cassiæ Cortex ............ | 8 | 0 |
| Catechu Extractum ........ | 2 | 3 |
| Cetaceum ................ | 3 | 0 |
| Cera flava, 3s. 6d. alba .... | 3 | 6 |
| Cerata, from 2s. 6d. to .... | 4 | 0 |
| Cinchonæ cordif. Cort. ...... | 9 | 0 |
| ——— lancif. Cort. ...... | 13 | 0 |
| ——— oblongif. Cort. .... | 16 | 0 |
| Cinnamomi Cortex ........ | 17 | 0 |
| Coccus (Coccinella) ........ | 60 | 0 |
| Colocynthidis ............ | 8 | 0 |
| Copaiba ................ | 7 | 0 |
| Cornu usti .............. | 1 | 0 |
| Cretæ ppt. .............. | 0 | 8 |
| Croci stigmata .......... | 64 | 0 |
| Cupri sulphas ............ | 1 | 0 |
| Cuprum ammoniatum ...... | 6 | 0 |
| Curcumæ Pulvis .......... | 2 | 6 |

CONFECTIO Aromatica, 8s. 6d.—Aurantii, 2s. 9d.—Opii, 4s. 6d.—Rosæ Gallicæ, 1s. 10d.—Rosæ Caninæ, 2s. —Scammoniæ, 15s. 0d.—Sennæ, 2s. —Amygdalæ, 5s. 0d.

EMPLASTRA Ammon. 8s. 0d.—Cumini, 2s.—Galbani Comp. 2s. 6d.—Lyttæ, 6s. 6d.—Opii, 2s. 6d.—Plumbi, 1s. 6d. —Resinæ, 1s. 6d.—Roborans, 2s. 0d. —Saponis, 2s. 6d.—Hydrarg. 2s. 6d. —Hydrar. cum Amm. 6s. 0d.

EXTRACTA Anthemidis, unc. 1s. 0d. —Belladon. 1s. 0d.—Cinch. 3s 0d.— Cinch. resinos. 4s. 0d.—Coloc. 2s. 0d. —Coloc. comp. 1s. 0d.—Conii, 0s. 6d. —Gent. 0s. 5d.—Glycyr. lb. 3s 0d.— Cascar. unc. 3s. 0d.—Hæmatoxyli, 0s. 5d.—Humuli, 0s. 9d.—Hyoscyami, 1s. 0d.—Jalapæ, 1s. 6d. Res. 2s. 8d. —Opii aquos, 3s. 8d.—Opii resinos, 3s. 0d.—Papaveris, 0s. 6d.—Rhæi, 2s. 4d.—Sarsapar. 10d.—Taraxaci, 6d.

| | s. | d. |
|---|---|---|
| Ferri carbon. 1s. 0d. sulph. lb. | 1 | 0 |
| Ferrum Ammoniatum ...... | 3 | 6 |
| ——— Tartarizatum ...... | 4 | 0 |
| Galbani Gummi-resinæ .... | 8 | 6 |
| Gentianæ Radix .......... | 1 | 3 |
| Guaiaci Resina ............ | 7 | 6 |
| Hydrargyrus purificatus .... | 5 | 4 |
| ——— cm Creta ...... | 4 | 0 |
| Hydrargyri Oxymurias ...... | 8 | 0 |
| ——— Submurias ...... | 8 | 6 |
| ——— Hydros. | 10 | 0 |
| ——— Nitrico-Oxydum .. | 8 | 6 |
| ——— Oxydum Rubrum | 8 | 0 |
| ——— Sulphuretum Nig. | 3 | 6 |
| ——— Præcipitatus Alb. | 8 | 0 |
| Hellebori nigri Radix ...... | 2 | 6 |
| Ipecacuanhæ Radix ........ | 20 | 0 |
| Jalapæ Radix .............. | 6 | 6 |
| Kino .................... | 12 | 0 |
| Liquor Plumbi Acetatis M. lb. | 1 | 0 |
| ——— Ammoniæ .... P.L. | 2 | 8 |
| ——— Vol. Corn. Cerv. .... | 1 | 3 |
| Linimentum Camphoræ comp. | 5 | 6 |
| ——— Saponis comp. .. | 5 | 6 |
| Lichen Islandicus ...... P. lb. | 1 | 9 |
| Lini Semina .............. | 0 | 6 |
| Lyttæ .................... | 11 | 0 |
| Magnesia ................ | 8 | 6 |
| ——— Carbonas ........ | 3 | 0 |
| ——— Sulphas ........ | 1 | 2 |
| Manna optima ............ | 6 | 0 |
| Mel Rosæ ................ | 2 | 6 |
| Moschus pod, 24s. in gr. unc. | 40 | 0 |
| Mastiche .............. lb. | 5 | 6 |
| Myristicæ Nuclei .......... | 25 | 0 |
| Myrrha elect. ............ | 6 | 6 |
| Opium (Turkey) .......... | 26 | 0 |
| Oleum Amygdalæ ...... lb. | 4 | 4 |

| | | |
|---|---|---|
| Oleum Anisi .......... unc. | 2 | 0 |
| —— Anthemidis........... | 5 | 0 |
| —— Cinnamomi Cassiæ .... | 8 | 0 |
| —— Caryophili .......... | 5 | 0 |
| —— Cajuputi ............ | 8 | 0 |
| —— Carui ............... | 1 | 9 |
| —— Juniperi ............ | 0 | 9 |
| —— Lavandulæ ........... | 4 | 0 |
| —— Lini .......... cong. | 8 | 6 |
| —— Menthæ piperitæ unc. | 4 | 0 |
| ———— viridis ...... | 3 | 0 |
| —— Olivæ, from 14*s*. 0*d*. to | 24 | 0 |
| —— Origani..........unc. | 2 | 0 |
| —— Pimentæ ............. | 4 | 6 |
| —— Ricini, from 7*s*. 0*d*. to | 10 | 0 |
| —— Rosmarini ...... unc. | 0 | 9 |
| —— Succini .. 2*s*. 4*d*. rect. | 2 | 6 |
| —— Sulphuretum ......lb. | 1 | 6 |
| —— Terebinthinæ rectif. .. | 2 | 0 |
| Oxymel Scillæ ............ | 3 | 0 |
| Papaveris Capsulæ (per 100) | 2 | 4 |
| Plumbi Carbonas ........ lb. | 0 | 8 |
| —— Superacetas ........ | 2 | 2 |
| Potassa Fusa ........... unc. | 0 | 6 |
| —— cum Calce ........ | 1 | 6 |

Potassæ—Nitras, lb. 1s.—Acetas, 8s. —Subcarbonas, 1s. 2d.—Carbonas, 4s. —Sulphas, 1s.—Sulphuretum, 1s. 6d. —Tartras, 3s.—Supertartras, 1s. 9d.

| | | |
|---|---|---|
| Pilulæ Hydrargyri ........ | 6 | 0 |
| —— Aloes comp.......... | 8 | 0 |
| ———— cum Colocynth. | 16 | 0 |
| —— Scillæ Compos. .... | 6 | 6 |
| —— e Styrace .......... | 48 | 0 |
| Piper longum et Nigrum .. | 2 | 6 |
| Pimento ..............lb. | 2 | 4 |
| Pulvis Aloes cum Canella .. | 6 | 6 |
| ———— Compositus .... | 10 | 0 |
| —— Antimonialis ........ | 7 | 0 |
| —— Cinnamomi Comp. .. | 16 | 0 |
| —— Cornu cervi cum Opio | 14 | 0 |
| —— Cretæ Composit. .... | 8 | 0 |
| ———— cum Opio | 8 | 0 |
| —— Kino Compositus .... | 12 | 0 |
| —— Scammoniæ Comp. unc. | 2 | 6 |
| —— Sennæ Composit. .. lb. | 8 | 0 |
| —— Tragacanthæ Comp. .. | 5 | 0 |
| Resina Flava et Nigra ...... | 0 | 5 |
| Rosæ Folia ............... | 7 | 6 |
| Ras Quassiæ .............. | 1 | 6 |
| Rhæi Radix (Russia) ...... | 30 | 0 |
| ———— (East India) .... | 14 | 0 |
| Sapo (Spanish)............ | 2 | 6 |
| Sarsaparilla Radix Inc....... | 5 | 0 |
| Scammoniæ Gum-Resina unc. | 2 | 6 |
| Scilla recens, 1*s*. 3*d*. siccata.. | 5 | 0 |
| Sennæ Folia Opt. Alexand. .. | 6 | 6 |
| Serpentariæ Radix.......... | 30 | 0 |
| Simaroubæ Cortex ........ | 4 | 0 |
| Sodæ Boras .............. | 4 | 0 |
| —— Carbonas ............ | 7 | 0 |
| —— Subcarbonas.......... | 1 | 4 |
| Sodæ Sulphas ............ | 0 | 8 |
| —— Phosphas ............ | 2 | 8 |
| Soda tartarizata ........ lb. | 2 | 4 |
| Spongia usta ..........unc. | 1 | 3 |
| Spiritus Ammoniæ.... M. lb. | 3 | 6 |
| ———— aromaticus | 4 | 6 |
| ———— fœtidus .. | 3 | 6 |
| ———— succinatus | 4 | 6 |
| —— Cinnamomi ver. .... | 3 | 6 |
| —— Juniperi Compos. .. | 3 | 0 |
| —— Lavandulæ ........ | 4 | 6 |
| ———— Compos. | 5 | 0 |
| —— Myristicæ ........ | 3 | 0 |
| —— Pimentæ .......... | 3 | 0 |
| —— Rosmarini ........ | 3 | 0 |
| —— Ætheris Aromaticus | 6 | 0 |
| ———— Compositus | 6 | 0 |
| —— Nitrici sp. gr. 860 | 5 | 6 |
| ———— Sulphurici | 6 | 0 |
| —— Vini rectificatus .... | 24 | 0 |
| Syrupi, from 1*s*. 8*d*. to .... | 3 | 0 |
| Sulphur.................... | 0 | 8 |
| —— Præcipitatum ...... | 1 | 0 |
| Tamarindus opt. ........... | 2 | 6 |
| Terebinthina Vulgaris ...... | 0 | 9 |
| ———— Canadensis .... | 8 | 0 |
| ———— Chia........... | 6 | 0 |
| Tragacantha Gummi........ | 10 | 0 |

Tincturæ—Aurantii, M. lb. 3s. 9d.—Aloes, 4s. 0d.—Aloes Comp. 9s. 6d.—Assafœt. 5s 6d.—Balsa. Tolut. 5s. 6d. —Benzoini Comp. 6s. 9d.—Camph. Comp. 4s. 0d.—Cardam. 4s. 0d.—Cardam. Comp. 4s. 0d.—Calum. 3s. 9d. Capsici, 3s. 9d.—Cascar. 4s 6d.—Cast. 7s. 0d.—Catechu, 4s. 0d—Cinchonæ, 6s. 0d—Cinch. Comp. 6s. 0d.—Cinch. Am. 7s 0d.—Croci, 6s. 6d.—Digitalis, 4s. 6d.—Ferri Ammon. 4s. 0d.—Cinnam. 4s. 6d.—Cin Comp. 4s. 6d. Gent. Comp. 4s. 0d.—Guaiaci, 6s 0d. —Guaiaci Am. 6s. 0d.—Helleb. Nig. 4s. 6d.—Humuli, 5s. 0d.—Hyoscyami Nig. 4s. 6d.—Jalap. 4s. 6d.—Japon. 4s. 6d.—Fer. Mur. 5s. 0d.—Kino, 5s. 0d.—Lyttæ, 3s. 9d.—Myrrhæ, 4s. 6d.—Opii, 7s. 0d.—Opii Camph. 4s 6d.—Quassiæ, 3s 9d.—Rhei, 4s. 6d. —Rhei Comp. 4s. 0d—Scillæ, 4s. 6d. —Sennæ, 4s. 9d.—Serpent. 6s. 0d.—Valer. 4s. 6d.—Valer. Am. 5s. 6d.—Zingiberis, 4s. 6d.

| | | |
|---|---|---|
| Valerianæ Radix ........... | 1 | 8 |
| Veratri Radix ............. | 1 | 6 |

Unguenta—Hydrar. fort, 5s. 0d.—Hydrar. Mit. 3s. 0d.—Hydrar Nitratis, 3s 3d.—Hydrar. Nitrico-oxy 2s. 9d. —Sulph. Comp. 1s. 9d.—Alia ung. 1s. 8d. to 2s. 0d.

| | | |
|---|---|---|
| Uvæ Ursi Folia.............. | 2 | 4 |

Vina—Aloes, 3s. 6d.—Antimoniale, 3s. 0d.—Ferri, 3s 6d.—Ipecac. 5s. 6d. —Opii, 8s. 0d.

| | | |
|---|---|---|
| Zinci Oxydum .............. | 4 | 6 |
| —— Sulphas purif. ...... | 2 | 0 |
| Zingiberis Radix opt. .... | 2 | 4 |

Leeches, 24s. per hundred.—Essential Salt of Lemons, 4s. 6d. per dozen.

